# An exploration of the link between adult attachment and problematic Facebook use

**DOI:** 10.1186/s40359-018-0245-0

**Published:** 2018-08-10

**Authors:** Sally Flynn, Chris Noone, Kiran M. Sarma

**Affiliations:** Risky and Extreme Behaviours Research Group (REX-GROUP), School of Psychology, National University of Ireland Galway, Galway, Ireland

**Keywords:** Facebook, Attachment, Psychological distress, Self-esteem

## Abstract

**Background:**

Previous studies have reported on positive and negative psychological outcomes associated with the use of social networking sites (SNSs). Research efforts linking Facebook use with depression and low self-esteem have indicated that it might be the manner in which people engage with the site that makes its use problematic for some people. The aim of the current study was to test a theoretical model of problematic Facebook use, using adult attachment style as the predictor variable of interest.

**Method:**

A cross-sectional design was employed wherein adult Facebook users (*n* = 717) completed measures of psychological distress, self-esteem, and adult attachment, in addition to measures of problematic Facebook use (i.e. social comparison, self-disclosures, impression management, & intrusive Facebook use). Data were analysed using hierarchical multiple regression and mediation analyses.

**Results:**

The results of this study indicated that attachment anxiety was predictive of all facets of problematic Facebook use, and that attachment avoidance was predictive of impression management, and social consequences of intrusive Facebook use. Further analyses confirmed the mediating influences of psychological distress and self-esteem on these relationships.

**Conclusions:**

Users of Facebook with higher levels of attachment insecurity may be gravitating towards the site in order to fulfil their attachment needs. This tendency is likely to be particularly prevalent for those individuals with low self-esteem who are experiencing psychological distress.

## Background

Large and diverse networks of people are embracing the use of social networking sites (SNSs). Recent demographic reports have indicated that engagement with SNSs is being adopted by increasing numbers of adults across the lifespan [1, 2]. Without question, users are deriving benefits from these sites, as evidenced by their continued growth and popularity [3]. This assertion also has scientific support, with many studies purporting positive psychological outcomes associated with the use of SNSs, including enhanced self-esteem [4], increased social capital [5], identity formation [6], self-expression [7], and cognitive benefits related to executive functioning [8].

Engagement with SNSs can be considered problematic when their use is associated with negative psychological outcomes. Increased loneliness [9] and anxiety [10, 11], and decreased self-esteem [12] are just some of the outcomes to be linked to SNS use in recent years. However the most contentious and often debated relationship concerns that of Facebook use and depression. Undoubtedly, a relationship exists between these two variables, as reported by a range of cross-sectional studies conducted in the area [13–16], yet despite this, the nature and direction of the relationship remains unclear.

A recent systematic review conducted in the area has identified four patterns of engagement with Facebook that are associated with depression; these are use of Facebook to engage in 1) social comparison, 2) impression management, 3) self-disclosures, and 4) intrusive use of Facebook (Flynn S, Summerville S, Sarma K: What is the *real *relationship between Facebook use and depression?, in preparation). A number of additional studies have also been identified which have found each of these patterns of responding to be associated with decreased self-esteem in some users of Facebook [12, 17, 18]. See Table 1 for an overview of these patterns of problematic Facebook use.

**Table 1:** Overview and description of dependent variables of interest in the current study

Facebook Behaviour	Description	Author (s)	Psychological correlate
Social comparison	Comparison of self in relation to others (may be an unconscious process) *(*e.g. *compulsively viewing photos or timeline content of another Facebook user (known or unknown)*	[16] Steers et al. (2015) [20] Appel et al. (2014) [21] Feinstein et al. (2013) [22] Tandoc et al. (2015)	Low mood/depression
Impression management	Deliberate concealment of certain aspects of the self in order to present a positive self-image *(*e.g. *applying filters to photo uploads to maximize attractiveness)*	[15] Rosen et al. (2013) [17] Mehdizadeh (2010)	Low mood/depression Self-esteem
Self-disclosure	Over-sharing of personal information on ones Facebook profile *(*e.g. *disclosing details of relationship conflict with partner)*	[23] Moreno et al. (2012) [24] Settani & Marengo (2015) [12] Forest & Wood (2012)	Low mood/depression Self-esteem
Intrusive Facebook use	Use of Facebook resulting in interruption to daily activities *(*e.g. *use of Facebook impacting academic studies)*	[13] Koc & Gulyagci (2013) [25] Blachnio et al. (2015) [26] Malik & Khan (2015) [18] Blachnio et al. (2016)	Low mood/depression Self-esteem

Consistent with the suggestions of [19], the authors of this review argued that it is the manner in which people engage with Facebook relative to use of the site in general (e.g. as typically measured by time spent online), that is associated with negative outcomes in some users.

However, the majority of studies that informed the findings of this review implemented cross-sectional methodologies, thus limiting the conclusions that can be drawn from these findings.

The results of these cross-sectional studies can be interpreted in three ways; 1) problematic Facebook use is affecting the mood and self-esteem of some users of the site, 2) some users of the site, who are experiencing low mood and low self-esteem feel driven to use Facebook in problematic ways as a means of coping with their emotional state, or 3) experiences of low mood and self-esteem drive people to use Facebook in problematic ways, and this subsequent use either further increases difficulties, or maintains low mood and self-esteem at their current levels. This paper argues that all three explanations are limited in not adopting a more comprehensive theoretical approach to understanding problematic Facebook use.

It is important for researchers to identify the psychological predictors of Facebook use, particularly when this use is associated with negative outcomes. Theoretical approaches regarding the general use of social networking platforms suggest that desire for belonging [27], enhancement of connections [5, 28, 29], and facilitation of self-presentations [30, 31] are some of the factors implicated in peoples’ decisions to embrace SNSs, generally. However, the psychological predictors of problematic Facebook use are less clear, and warrant investigation by prospective researchers.

Given that SNSs embody social behaviour and interpersonal relating, attachment theory is proposed as a novel theoretical approach to enhance our understanding of problematic Facebook use. Attachment theory [32–35] posits that individuals are born with an innate desire to form affectional ties with others, and that this drive for human connection persists across the lifespan. In infancy, attachment behaviours, designed to elicit contact and comfort from caregivers, are instinctual and can comprise crying, reaching, cooing, smiling, and sucking. Throughout the lifespan, individuals continue to act in ways that will elicit contact and connection from others, though these specific behaviours can vary according to a person’s individual attachment profile. We argue here that certain people gravitate towards Facebook in order to meet their attachment needs, and that this engagement becomes problematic due to the complex profiles of attachment insecurity. Though childhood attachment is typically discussed in relation to specific categorical styles, adult attachment is best considered to lie amongst two continuums - that of attachment anxiety and attachment avoidance [36]. High levels of attachment anxiety are associated with increased dependency [37], preoccupation with the availability of others [38], emotionally lability [39], and self-deprecation [40], whereas high levels of attachment avoidance manifest in resistance with intimacy [41], inhibition of emotional expression [42], and a strong sense of independence and self-reliance [36]. With regards to adult attachment, low levels of anxiety and avoidance are thought to be reflective of attachment security [43].

One of the most studied phenomena in relation to attachment theory concerns the hypothetical construct of internal working models (IWMs; [33, 35]). These internalised mental representations are thought to be informed by the quality of early parent/child interactions [44]. IWMs consist of two complementary models of the self and others, which are thought to guide thoughts, feelings, and behaviour across the lifespan [33]. For example, through repeated, consistent, and positive interactions with their primary caregiver, a child may well come to view others in their lives as safe, reliable, and dependable, and themselves as worthy of care and love. Conversely, a child who has experienced inconsistent and unpredictable interactions with their caregiver may develop a view of others as being untrustworthy or unavailable, and a view of themselves as being unworthy of love and attention. These internal representations are thought to be reworked across the lifespan, thus impacting on a person’s view of themselves, and those whom they encounter in their social world [33, 35]. The authors argue that self-esteem may offer the closest insight into the hypothetical and somewhat invisible construct of IWMs, given their relevance to how people view themselves and how others respond to them.

Operating outside of conscious awareness [45, 46], IWMs are considered to provide organizational structure to the attachment system, thus having implications for how individuals respond to threats to their attachment system via emotional regulation. When threatened, the attachment system of those with high levels of attachment anxiety can become hyper-activated, resulting in exaggerated or heightened displays of emotion [47], and decreased confidence in the self management of distress [38]. For individuals with high levels of attachment avoidance, de-activation of the attachment system can occur, resulting in defensive responding through the suppression or denial of overt emotional distress [48, 49]. Given the importance of IWMs and emotional regulation within attachment theory, the relationship between attachment insecurity and problematic Facebook use will be further explored by considering the mediating influences of psychological distress and self-esteem.

Previous research has applied attachment theory to social networking contexts. However, notwithstanding the fact that these studies suffered from a number of methodological limitations, their focus tended towards Facebook engagement in general [50], such as time spent online [51], and positive facets of SNS use, including the derivability of social capital [52], and intimacy [53]. A recent study that examined the relationship between adult attachment and use of Facebook provided tentative support for the assertion that attachment might predict problematic engagement with the site, by concluding that individuals characterised by attachment insecurity engage in a greater use of Facebook following emotional distress [54].

**Fig. 1:**
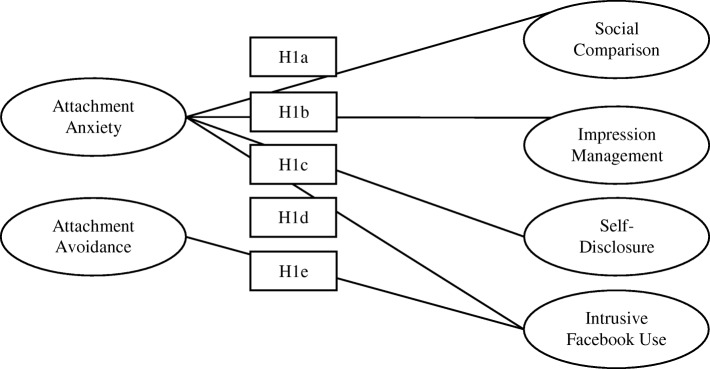
Graphical illustration of H1 in the current study

**Fig. 2:**
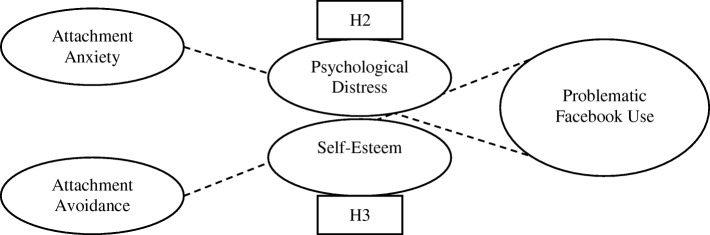
Graphical illustration of H2 and H3 in the current study

The current study tests a theoretical model of problematic Facebook use, focusing on adult attachment as the main predictor variable and psychological distress and self-esteem as potential mediating influences. It focuses on four patterns of engagement with Facebook that have been evidenced to be problematic, thus offering a clinically meaningful insight into problematic Facebook use in the general population. Given the tendency to engage in attachment focused hyper-activating strategies, a preoccupation with others, and the strong need for belonging and acceptance, individuals with high attachment anxiety are expected to engage more frequently in all facets of problematic Facebook use. Given the tendency to engage in attachment focused deactivating strategies, and a resistance towards intimacy and dependence, individuals with high levels of attachment avoidance are expected to engage in aspects of intrusive Facebook use, as the site offers less threatening and less intimate forms of interaction with others. The study tests three hypotheses, which are presented graphically in Fig. 1 (Graphical Illustration of H1 (a-e) in the Current Study) and Fig. 2 (Graphical Illustration of H2 and H3 in the Current Study).

H1 – Engagement in problematic Facebook use will be predicted by higher levels of attachment insecurity. Specifically;

- H1a – Engagement in social comparison on Facebook will be predicted by higher levels of attachment anxiety.
- H1b – Engagement in impression management on Facebook will be predicted by higher levels of attachment anxiety.
- H1c- Engagement in self-disclosures on Facebook will be predicted by higher levels of attachment anxiety.
- H1d- Engagement in intrusive use of Facebook (i.e. use resulting in 1) social consequences, 2) emotional consequences & 3) impulsive/risky use) will be predicted by higher levels of attachment anxiety.
- H1e- Engagement in intrusive use of Facebook (i.e. use resulting in 1) social consequences, and 2) impulsive/risky use) will be predicted by higher levels of attachment avoidance. Emotional consequences were not anticipated to be problematic for those with high levels of attachment avoidance, due to the tendency for these individuals to suppress or deny emotional distress [48, 49].

H2 – Psychological distress will mediate the relationship between attachment insecurity and engagement in problematic Facebook use.

H3 – Self-esteem will mediate the relationship between attachment insecurity and engagement in problematic Facebook use.

## Method

### Design

The current study implemented a cross-sectional design, in which adult Facebook users completed an online survey that gathered responses on psychological distress, self-esteem, attachment, and their specific use of Facebook.

### Participants

Participants were subscribers to the SNS Facebook, who were recruited online via Facebook, Twitter, and LinkedIn, using an exponential, non-discriminative, virtual snowball sampling method. Within the context of the current study, the primary author provided a brief written overview of the study, along with a hyperlink to an external survey host website, which were posted on her personal Facebook page. A request was made for participants to share the hyperlink to their own Facebook page, once they had completed the online measures. Non-probability sampling was used in this study due to the ease of access of the study population via the social networking site, Facebook. The authors hoped this would increase the geographical scope and quantity of participants reached, so as to increase the representativness of the current sample. Inclusion criteria required respondents to be 18 years or over. A total of 1275 participants followed the hyperlink to the survey, 1094 of whom commenced the survey by indicating their consent and providing some demographic information. Of these, 65.5% completed the survey in its entirety, resulting in a complete data set of 717 participants. A Pearson *X*^*2*^ test indicated that survey completers and non completers did not differ significantly from one another with respect to age (*p* = *.*06) and gender (*p* = .16). An independent samples t-test found that survey non-completers had a significantly higher number of Facebook friends (*m* = 460, *sd* = 411), relative to survey completers (*m* = 370, *sd* = 339; *t* (625) = − 3.58), *p* < .01). Becoming distracted by greater amounts of social content or a higher frequency of communication attempts from Facebook friends may offer some explanation with respect to the variation in survey completion between these groups.

The sample consisted of 137 (19.1%) males and 578 (80.6%) females, aged 18–65 (M = 31, SD = 8.40). This gender imbalance in the demographic profile of SNS subscribers is a commonly observed trend amongst SNSs in general, and Facebook, specifically [1]. Seventy nine percent of participants were Irish, 12% were British, 3% were Australian, 3% were European, 2% were American, and 1% was Canadian. Forty-three percent of participants were in a relationship, 29% were single, and 28% were married. Eighty nine percent of the sample had completed or were completing third level education, and 11% had completed second level education. In terms of Facebook usage characteristics, the majority of participants reported using two different devices to access Facebook (44.6%) and being somewhat engaged with the social networking platform (52.4%). Participants, on average, had 370 Facebook friends, and reported spending 72 min on Facebook per day.

### Measures

#### Information sheet

An information sheet was first presented to participants, describing the nature of the study, matters relating to consent, and any potential risks to taking part.

#### Consent form

Prior to accessing the survey, a consent form was administered to participants, which summarized the main information that was pertinent to their involvement in the study.

#### Demographic questionnaire

A brief questionnaire was used to obtain demographic information from participants relating to age, gender, relationship status, and education level. Participants were also asked for specific information relating to their use of Facebook.

#### Self-esteem

The Rosenberg Self Esteem Scale (RSES; [55]) is a ten item questionnaire, consisting of both positive (e.g. *“On the whole I am satisfied with myself)* and negative (e.g. “*At times, I think I am no good at all”*) self-statements. Participants were required to indicate their level of agreement with each item from the following four response options (i.e. “*strongly agree”, “agree”, “disagree”, “strongly disagree”).* The RSES is amongst the most widely used measures of self-esteem [56]. demonstrating excellent internal reliability across several countries [57]. Cronbach’s alpha for the RSES in the current study was .74. Convergent validity of the scale has been confirmed via correlations between the RSES and additional measures of self-esteem [58, 59].

#### Psychological distress

The Depression Anxiety and Stress Scale (DASS; [60]), is a 42-item questionnaire consisting of three, 14-item self-report scales, measuring anxiety (e.g. *“I feared that I would be thrown by some trivial but unfamiliar task”*), depression (e.g. *“I felt that life was meaningless”*), and stress or tension (e.g. *“I tended to over-react to situations”*). Participants were required to indicate on a four-point Likert scale ranging from *0* = “*did not apply to me*” to *3* “*applied to me very much or most of the time*”, the extent to which a series of statements applied to them over the past week. The DASS is a widely used measure of psychological distress in both community and clinical samples, and the scale demonstrates good discriminant validity [60]. When scored as a unidimensional measure of psychological distress, the DASS showed excellent reliability (α = 0.96). Adequate reliability for each of the subscales was also demonstrated (i.e. anxiety α = 0.87, depression α = 0.95, stress α = 0.92). Convergent and discriminant validity of the DASS have been established in previous research that has correlated the scale with established measures of anxiety, depression, and positive and negative affect [61].

#### Social comparison

The Iowa Netherlands Comparison Orientation Measure (INCOM; [62]), has been used to measure social comparison orientation in both adults and adolescents. An adapted version of the INCOM was used in the current study to measure participants’ social comparison tendencies on Facebook. Adaptation was based upon previous research carried out in this area [16], and consisted of the following modifications for each item (e.g. “*I often consider how I am doing socially with how others are doing*” ➔ “*When I am on Facebook, I often consider how I am doing socially with how others are doing)*. Participants were required to indicate by means of a five-point Likert scale ranging from 1 = “*disagree strongly”* to 5 = “*agree strongly”*, the extent to which they agreed with a number of statements regarding social comparison. The scale consists of 11 items, with higher scores indicating greater levels of social comparison. Cronbach’s alpha for the INCOM in the current study was 0.84. Previous research has indicated moderate correlations between the INCOM and the Social Comparison Motive Scale (SCMS), thus providing support for the discriminant and convergent validity of the scale [63].

#### Impression management

The Perception of False-Self Scale (POFSS; [64]) was initially developed to determine false self-perceptions in an adolescent population. An adapted version of the POFSS was used to measure participants’ tendencies to engage in impression management on Facebook. Adaptation was informed by similar research that had been conducted in relation to impression management on Facebook, using an adult population [65]. The scale consists of 21 items and participants were required to indicate which of five response options (i.e. “*strongly disagree”, “disagree”, “neither agree nor disagree”, “agree’, & “strongly agree”*) best described the extent to which they presented their true selves on Facebook (α = 0.86). Significant correlations between the POFSS and additional measures of self-perception have provided support for the convergent validity of the scale [64].

#### Facebook self-disclosure

Five bespoke items were created by the researcher to capture participants’ tendencies to share information of a personal nature on their Facebook profiles. The development of these items was informed by previous research in the area, which indicated that disclosures on SNSs could be positive, negative or neutral [66]. Participants were required to indicate by means of a five-point Likert scale ranging from 0 = *“never*” to 4 = *“very often”,* the frequency with which they engaged in certain self-disclosures on Facebook (e.g. *How often do you share information about your mood states (*e.g. *anger, sadness, frustration) on Facebook, that you would not usually share in front of large groups of people when you are offline?*) Factor analysis was completed on the Facebook self-disclosure items, supporting a one-factor solution for measuring participants’ tendencies to disclose on Facebook, which accounted for 49.9% of the total variance. The Kaiser-Meyer-Olkin measure of sampling adequacy supported the adequacy of the analysis (KMO = .803) and Bartlett’s test of Sphericity was significant (*p* = .000).The self-disclosure scale showed adequate reliability in the current study (α = 0.77).

#### Intrusive Facebook use

The Problematic and Risky Internet Use Screening Scale (PRIUSS) was developed by [67] to measure adolescents’ problematic Internet use. The scale was adapted for use in the current study to obtain a measure of participants’ intrusive Facebook use. Adaptation involved substituting the term ‘Internet’ for ‘Facebook’ for each of the 18 items in the scale (e.g. “*how often do you skip out on social events to spend time on the Internet*” changed to “*how often do you skip out on social events to spend time on Facebook*”). Participants were required to indicate by means of a five-point Likert scale ranging from 0 = “*never”* to 4 = “*very often”*, the extent to which their use of Facebook resulted in undesirable outcomes. The scale consists of three subscales that provide a measure of 1) social consequences related to Facebook use (α = 0.64), 2) emotional consequences related to Facebook use (α = 0.87), and 3) risky and impulsive Facebook use (α = 0.89). The construct validity of the scale has been confirmed previously by correlating each of the subscales with participants’ reports of daily hours spent on the Internet [67].

#### Adult attachment

The Experiences in Close Relationships-Revised Questionnaire (ECR-R; [43]) is a revised version of the ECR [68]. The 36-item questionnaire provides a linear measure of adult attachment dimensions. It consists of 18 items pertaining to attachment anxiety (e.g. “*I am afraid that I will lose my partner’s love*”) and 18 items relating to attachment avoidance (e.g. “*I get uncomfortable when my partner wants to be very close*”). Participants were required to indicate on a seven-point Likert scale ranging from 1 = “*strongly disagree’* to 7 “*strongly agree*”, the extent to which they agreed with each statement. Lower scores on each subscale are considered to be indicative of attachment security. For the current study, participants were encouraged to complete this measure by considering how they felt in close relationships in general (e.g. with romantic partners, close friends or family members), relative to with romantic partners specifically. The term “partner” was substituted with “close relationship partner” for each item in order to facilitate accurate responding. The ECR is the preferred scale of choice amongst adult attachment researchers [40], and has demonstrated excellent reliability estimates [69]. In terms of validity, scores on the ECR-R have accounted for 30–40% of the variance in participants’ diary ratings of attachment-related emotions during social interactions [70]. In the current study, Cronbach’s alphas for the anxiety and avoidance subscales were 0.95 and 0.94 respectively, and correlation between the two scales was 0.59.

#### Distractor items

A number of distractor items (e.g. “*I like to change my profile picture on Facebook”*) were also incorporated into each of the Facebook measures in order to ensure that participants did not become aware of the purpose of the questions, and modify their responses as a result.

### Procedure

Advertisements regarding the study and a link to the survey website was posted to the researcher’s personal Facebook, Twitter (http://www.twitter.com), and Linkedin (http://www.linkedin.com) profiles, and shared via a snowball sampling method. Participants who clicked on the hyperlink were directed to an external survey host website. Here they were presented with information regarding the study and information relating to consent. Participants were requested to open their Facebook profiles in a separate window whilst completing the measures in order to ensure more accurate recall of online behaviours, which they may have been unaware of. Participants were able to navigate their way through the survey interface by clicking on a ‘Next’ button at the bottom of each page. Participants were not permitted to proceed to the next questionnaire until they had answered all items. This is a benefit of online surveys of this type and served to reduce the amount of missing data in the current study. However, inadvertently, this option was not applied to responses on the anxiety subscale of the ECR-R, which resulted in a small amount of missing data.

## Results

### Statistical strategy

#### Data preparation

Data was screened for missing data prior to analyses. A small amount of missing data (i.e. 3.4%) was observed for the anxiety subscale on the ECR-R. The Expectation Maximization (EM) algorithm [71] was applied to impute the missing data (MCAR = 0.544). A transformation algorithm was applied in order to reverse code items on the INCOM, POFSS, and ECR-R. The three-factor version of the DASS was used to test H1. However, for parsimony of findings, a higher order factor of global psychological distress was used in the subsequent mediation analysis to test H2 & H3. A higher order factor of global psychological distress on the DASS has been confirmed in previous research [72]. Given the large sample in the current study (*n* > 300), and in line with recommendations from previous research, skewness values of > 2 and Kurtosis values of > 7 were used as cut-off points to determine violation of the assumption of normality [73]. Violation of this assumption was observed for scores on the Facebook Self-Disclosure Scale with Skewness of 2.17 (SE = 0.09) and Kurtosis of 7.10 (SE = 0.18). A Log10 transformation algorithm was applied in order to normalize this data for inferential analyses. Visual inspection of histograms and Q-Q-Plots, in addition to Skewness and Kurtosis values for each subscale on the DASS also indicated slightly skewed distributions (all *p*’s <.0005 ). However, this was expected given the positively skewed prevalence of depression, anxiety and stress in the normal population. Furthermore, according to [74], violation of the assumption of normality in larger samples is not expected to bias inferential analyses.

#### Analytic strategy

In preparation for hypotheses testing, Pearson’s zero order correlations were conducted, examining the associations between each of the predictor and criterion variables in the current study. Daily time spent on Facebook and number of Facebook friends were included in this analysis given previous links between these variables and negative psychological outcomes [75, 76]. Age and gender were also included in order to examine variations with respect to problematic Facebook use amongst these demographic variables. The results of this informed the completion of multiple linear regression analyses, which assisted in the development of predictive models of problematic Facebook use. As recommended by previous research, in an attempt to reach the most parsimonious solution, the number of predictor variables for this analysis was refined by retaining only those that significantly contributed to the models [77]. In order to test H1, a series of block-wise regression analyses were performed. Block 1 consisted of control variables, and Block 2 included the addition of attachment scores, as measured by the ECR-R. The final stage of analysis involved examining the mechanisms through which attachment insecurity influenced engagement in problematic Facebook use. This was achieved via the completion of a series of mediation analyses, using the PROCESS macro add on for SPSS (version 20), in which the causal effects of attachment insecurity on problematic Facebook use were interpreted by considering the mediating influences of psychological distress and self-esteem.

### Descriptive statistics

Table 2 presents descriptive statistics, with means and standard deviations (SDs) for each of the main psychological variables in the current study. Scores on each of the attachment dimensions were lower than what has been previously reported (see [78]), where age-matched norms of 3.56 and 2.97 have been reported for attachment anxiety and avoidance, respectively.

**Table 2:** Descriptive statistics, means and standard deviations for the main study variables

Variable	Mean	SD	Min	Max
Social comparison	29.13	7.93	11	52
Impression management	49.70	10.49	23	81
Self-disclosure	0.35	0.33	0	1.30
Intrusive - social	2.28	2.35	0	13
Intrusive - emotional	1.94	2.80	0	19
Intrusive - risky/impulsive	5.94	5.23	0	28
Attachment anxiety	2.75	1.23	1	7
Attachment avoidance	2.51	1.05	1	6.67
Stress	9.60	7.31	0	42
Depression	5.85	7.41	0	42
Anxiety	3.99	5.04	0	33
Self-esteem	20.39	4.09	13	30

Mean scores for self-esteem were consistent with those reported in a previous internationally representative, normative study [57]. Each subscale score on the DASS was also in line with previous norms reported in a large, non-clinical sample [60].

### Correlation analysis

The correlation matrices are presented in Tables 3 and 4. Given the relatively large sample in the current study, it is important to note that even small relationships between variables are likely to emerge as statistically significant. To reduce the likelihood of a Type 1 error, the authors focused on relationships that indicated a medium effect size (*r* > 0.30) or larger, and used an adjusted Bonferroni alpha level of 0.003. Significant correlations were observed between high levels of attachment anxiety and greater social comparison (*r* = 0.42), impression management (*r* = 0.42), social consequences of intrusive Facebook use (*r* = 0.37), and impulsive/risky Facebook use (*r* = 0.32). A relationship also emerged between high levels of attachment avoidance and greater impression management tendencies (*r* = 0.39). Significant, negative correlations emerged between attachment insecurity and self-esteem (i.e. attachment anxiety *r* = − 0.43, attachment avoidance *r* = − 0.35), whilst significant positive correlations were noted between attachment insecurity and psychological distress (i.e. attachment anxiety *r* = 0.49, attachment avoidance *r* = 0.33). Psychological distress and self-esteem also significantly correlated with social comparison, impression management, social consequences of intrusive Facebook use and impulsive/risky Facebook use (all *r*’s > ± 0.3). As expected, stress, depression, and anxiety scores all correlated significantly, and strongly with one another (all *r*’s > 0.7), indicating convergent validity amongst the DASS subscales.

**Table 3:** Correlation matrix of predictor and criterion variables in the current study

Variable	1	2	3	4	5	6	7	8	9	10	11	12	13	14	15	16	17
1. Social comparison	**–**																
2. Impression management	0.47**	–															
3. Self-disclosure	0.40**	0.21**	–														
4. Intrusive-social	0.45**	0.42**	0.43**	–													
5. Intrusive-emotional	0.39**	0.33**	0.36**	0.54**	–												
6. Intrusive-risky/impulsive	0.44**	0.40**	0.32**	0.53**	0.60**	–											
7. Attachment anxiety	0.42**	0.42**	0.26**	0.37**	0.25**	0.32**	–										
8. Attachment avoidance	0.22**	0.39**	0.19**	0.28**	0.17**	0.21**	0.59**	**–**									
9. Stress	0.37**	0.35**	0.26**	0.36**	0.22**	0.28**	0.43**	0.24**	–								

Note: ** p < .001

**Table 4:** Correlation matrix of predictor and criterion variables in the current study

Variables	1	2	3	4	5	6	7	8	9	10	11	12	13	14	15	16	17
10. Depression	0.33**	0.35**	0.21**	0.33**	0.16**	0.27**	0.47**	0.36**	0.72**	–							
11. Anxiety	0.33**	0.32**	0.26**	0.35**	0.23**	0.26**	0.42**	0.29**	0.76**	0.71**	–						
12. Total distress	0.38**	0.38**	0.27**	0.38**	0.22**	0.30**	0.49**	0.33**	0.92**	0.91**	0.86**	–					
13. Self-esteem	−0.26**	− 0.31**	−.012**	− 0.17**	− 0.15**	− 0.19**	− 0.32**	− 0.25**	−0.20**	− 0.19**	− 0.16**	− 0.20**	–				
14. Daily time on Facebook	0.11**	0.06	0.17**	0.24**	0.20**	0.32**	0.08	0.03	0.10**	0.12**	0.11**	0.12**	0–.06	–			
15.No. of Facebook friends	0.19**	0.10	0.13**	0.08	0.13**	0.22**	0.17**	0.09	0.10	0.06	0.11**	0.01	−0.03	0.15**	–		
16. Age	−0.19**	− 0.15**	−.00	− 0.11**	−0.09	− 0.22**	−0.14**	− 0.02	−0.11**	− 0.12**	− 0.13**	0–.13**	0.12**	−0.11**	0–.29**	–	
17. Gender	0.13**	.00	−.00	0.03	0.11**	0.08	−0.06	−0.17**	0.05	0–.05	.00	.00	−0.08	0.09	−0.02	−0.01	–

Note: ** p < .001

### Hypothesis testing

#### H1: Engagement in problematic Facebook use will be predicted by higher levels of attachment insecurity

As can be seen in Table 5, attachment avoidance emerged as a significant predictor of the social consequences of intrusive Facebook use, when the effects of additional predictors were controlled for; therefore H1e was partially supported. Though not originally hypothesised, attachment avoidance also emerged as a significant predictor of impression management in the current study.

**Table 5:** Hierarchical multiple regression analyses testing predictive models of attachment insecurity and problematic Facebook use

	Models	Self-disclosure			Intrusive-risky/impulsive			Social comparison			Impression management			Intrusive- social			Intrusive- emotional		
	Predictors	B	SE B	*β*	B	SE B	*β*	B	SE B	*β*	B	SE B	*β*	B	SE B	*β*	B	SE B	*β*
	Stress	0.01	.00	0.13*	0.12	0.04	0.17*	0.25	0.06	0.28**	0.30	0.07	0.21**	0.06	0.02	0.18*	0.03	0.02	.09
	Daily time	.00	.00	0.14**	0.02	.00	0.25**	.00	.00	0.03	.00	.00	.00	0.01	.00	0.19**	.01	.00	.15**
	No. of friends	9.35	.00	0.10*	.00	.00	0.12**	.00	.00	0.12**	.00	.00	0.04	2.11	.00	.00	.00	.00	.08*
	Self-esteem	−.00	.00	−0.07*	−0.14	0.04	−0.11*	−0.35	0.07	−0.18**	−0.64	0.09	−0.25**	− 0.06	0.02	− 0.10*	−0.07	0.03	−.10*
	Age	.00	.00	0.08*	−0.07	0.22	−0.19*	− 0.08	0.03	− 0.09*	−0.09	0.04	−0.07	− 0.01	0.01	− 0.03	−0.01	0.01	−.02
	Gender	−0.02	0.03	−0.02	0.53	0.43	0.04	2.10	0.66	0.12**	−0.56	0.88	− 0.02	−0.02	0.20	−.00	0.62	0.25	.09*
	Anxiety	0.01	.00	0.13*	0.06	0.05	0.06	0.16	0.08	0.10	0.22	0.19	0.11*	0.08	0.02	0.17*	0.07	0.03	.12*
1	R^2^		0.11**			0.21**			0.21**			0.19**			0.18**			0.10**	
	Stress	0.01	.00	0.13*	0.12	0.04	0.17*	0.24	0.06	0.22**	0.29	0.07	0.20**	0.06	0.02	0.18*	0.03	0.02	.08
	Daily time	.00	.00	0.14**	0.02	.00	0.25**	.00	.00	0.03	.00	.00	.00	0.01	.00	0.19**	0.01	.00	.15**
	No. of friends	9.35	.00	0.10*	.00	.00	0.12*	.00	.00	0.12**	.00	.00	0.02	−5.82	.00	−0.01	.00	.00	.07
	Self-esteem	−.00	.00	−0.05	−0.11	0.05	−0.08*	−0.30	0.07	−.016**	−0.48	0.09	−0.19**	−0.03	0.02	−0.06	− 0.05	0.03	−.07
	Age	.00	.00	0.07*	−0.08	0.22	−0.13**	−0.09	0.03	−0.10*	− 0.11	0.04	− 0.09*	−0.01	0.01	−0.05	− 0.01	0.01	−.03
	Gender	−.00	0.03	−0.01	0.86	0.44	0.07	2.53	0.67	0.13**	0.87	0.87	0.03	0.19	0.20	0.03	0.78	0.25	.11*
	Anxiety	0.01	.00	0.11	0.03	0.05	0.03	0.12	0.08	0.07	0.08	0.10	0.04	0.06	0.02	0.13*	0.05	0.03	.09
	Attachment avoidance	0.03	0.01	0.10*	0.67	0.18	0.13**	0.88	0.27	0.17**	2.88	0.35	0.29**	0.42	0.08	0.19**	0.32	0.10	.12*
2	R^2^ full model		0.12**			0.23*			0.22**			0.26**			0.21**			0.11**	
	R^2^ Change		0.01**			0.02*			0.01**			0.07**			0.03**			0.01*	
	Stress	0.01	.00	0.11	0.09	0.04	0.13*	0.17	0.05	0.16**	0.24	0.07	0.17*	0.05	0.02	0.14*	0.02	0.02	.05
	Daily Time	.00	.00	0.14**	0.02	.00	0.25**	.00	.00	0.02	.00	.00	.00	0.01	.00	0.19**	0.01	.00	.15**
	No. of friends	8.49	.00	0.09*	.00	.00	0.10*	.00	.00	0.10*	.00	.00	0.01	.00	.00	−0.02	.00	.00	.06
	Self-esteem	−.00	.00	−0.03	−0.08	0.05	−0.06	−0.23	0.07	−0.12**	−0.42	0.09	−0.16**	−0.02	0.02	0.03	−0.04	0.03	−.05
	Age	.00	.00	0.08*	−0.07	0.22	−0.12*	−0.07	0.03	−0.08*	−.010	0.04	−0.08*	0.01	0.01	0.04	−0.01	0.01	−.02
	Gender	−.00	0.03	−.00	0.86	0.44	0.07	2.52	0.65	0.13**	0.86	0.86	0.03	0.19	0.20	0.03	0.77	0.25	.11*
	Anxiety	0.01	.00	0.10	0.01	0.05	0.01	0.08	0.08	0.05	0.06	0.10	0.03	0.05	0.02	0.12*	0.05	0.03	.08
	Attachment avoidance	0.02	0.01	0.05	0.31	0.21	0.06	−0.13	0.31	−0.02	2.15	0.41	0.21**	0.24	0.09	0.11*	0.16	0.12	.06
	Attachment anxiety	0.03	0.01	0.12*	0.65	0.19	0.15*	−1.82	0.28	0.28**	1.32	0.37	0.16**	0.33	0.09	0.18**	0.29	0.11	.13*
3	R2 full model		0.12**			0.24**			0.27**			0.27**			0.23**			0.12**	
	R2 change		0.01**			0.01**			0.04**			0.01**			0.02**			0.01*	

*Notes:*

1 = Block 1 (contribution of control variables)

2 = Block 2 (additional contribution of attachment avoidance)

3 = Block 3 (additional contribution of attachment anxiety)

* *p* < .05, ** *p < .*001

Attachment anxiety emerged as a significant predictor of all aspects of problematic Facebook use, even when the effects of additional significant predictor variables had been controlled for, thus supporting H1 a-d (See Table 5). Attachment anxiety was the most frequent predictor of problematic Facebook use in the current study, featuring in all eight predictive models.

#### H2 & H3- psychological distress and self-esteem will mediate the relationship between attachment insecurity and problematic Facebook use

In order to determine whether psychological distress and self-esteem accounted for the observed relationships between attachment insecurity and problematic Facebook use, a series of mediation analyses were carried out using the PROCESS macro add on for SPSS (Version 20) [79]. Though not emerging as a significant predictor of all facets of problematic Facebook use, attachment avoidance was included in this analysis across all six problematic Facebook uses, in order to determine whether an indirect relationship would be observed via the mediating variables. This step was informed by recommendations from [80], who have argued that caution should be taken when allowing the absence of an X → Y relationship inform subsequent mediation analyses. Given that it emerged as a frequent predictor of problematic Facebook use in the previous regression analyses, daily time spent on Facebook was controlled for by entering this as a co-variate in the PROCESS macro. In line with recommendations from [79], bootstrapping techniques [81] were implemented, utilising 1000 bootstrap samples. Direct and indirect effects were considered statistically significant when the 95% confidence intervals for each model did not include zero. It was not possible to determine the size of the observed indirect effects as the use of the kappa-squared (k^2^) statistic has not yet been developed for use in models involving covariates [82].

The results of the full mediation analysis (See Table 6) indicated that there was a significant indirect effect of attachment avoidance across all facets of problematic Facebook use (with the exception of self-disclosures) that was mediated by higher levels of psychological distress, and low levels of self-esteem. The analysis also indicated that the relationships between attachment anxiety and problematic Facebook use (with the exception of emotional consequences of intrusive Facebook use) were significantly mediated by high levels of psychological distress, and that the relationships between attachment anxiety and social comparison, impression management, and risky/impulsive Facebook were significantly mediated by low levels of self-esteem. These findings suggest that individuals with high levels of attachment insecurity may be prone to engaging with Facebook in problematic ways due to low self-esteem, and that these relationships may be particularly heightened when experiencing psychological distress.

**Table 6:** Mediation analyses testing the influence of psychological distress and self-esteem on the relationships between attachment insecurity and problematic Facebook use^§^

	Effect of IV on MV (a paths)		Effect of MV on DV (b paths)		Direct effect (c^’^path)	Total effect (c path)	Indirect effects (a x b)				Indirect effects (a x b) 95% Confidence intervals			
Variables	M^1^	M^2^	M^1^	M^2^	M^1^ - M^2^	X-Y	M^1^	M^2^			M'^1^		M^2^	
	*b*	*b*	*b*	*b*	*b*	*b*	*b*	SE	*b*	SE	L	U	L	U
Attachment anxiety	6.98**	−1.05**												
Social comparison			0.09**	−0.26**	1.78**	2.68**	0.66	0.13	0.27	0.08	**0.41**	**0.93**	**0.14**	**0.45**
Impression management			0.12**	−0.48**	2.24**	3.61**	0.86	0.20	0.51	0.11	**0.49**	**1.25**	**0.31**	**0.74**
Self-disclosures			0.04**	.00	0.04**	0.07**	0.02	0.01	0.00	.00	**0.01**	**0.03**	.00	0.01
Intrusive - social			0.03**	−0.03	0.42**	0.67**	0.22	0.05	0.03	0.02	**0.14**	**0.32**	−0.01	0.06
Intrusive- emotional			0.02*	−0.05	0.38**	0.54**	0.12	0.06	0.05	0.02	.00	0.25	.00	0.10
Intrusive – risky/impulsive			0.04**	−0.10*	0.83**	1.25**	0.31	0.10	0.11	0.05	**0.13**	**0.54**	**0.02**	**0.21**
Attachment avoidance	5.57**	−0.95**												
Social comparison			0.14**	−0.35**	0.53**	1.63**	0.77	0.12	0.34	0.08	**0.56**	**1.07**	**0.20**	**0.54**
Impression management			0.15**	−0.52**	2.59**	3.89**	0.82	0.17	0.49	0.11	**0.53**	**1.17**	**0.30**	**0.72**
Self-disclosures			.00	.00	0.04**	0.06**	0.02	.00	.00	.00	.00	0.03	.00	0.01
Intrusive - social			0.04**	−0.04	0.37**	0.62**	0.21	0.05	0.04	0.02	**0.14**	**0.33**	**0.01**	**0.08**
Intrusive - emotional			0.02**	−0.06*	0.26*	0.44**	0.13	0.04	0.06	0.02	**0.06**	**0.23**	**0.02**	**0.11**
Intrusive – risky/impulsive			0.06**	−0.13**	0.53**	0.99**	0.34	.009	0.13	0.04	**0.20**	**0.54.**	**0.05**	**0.22**

^§^*Notes:* a: DV: Problematic Facebook use (i.e. social comparison, impression management, self disclosures, social, emotional consequences, risky/impulsive use)

M^1^: Psychological distress, M^2^: Self-esteem

IV: Attachment anxiety/Attachment avoidance

b:X = IV, Y = DV

c: SE = Bootstrapped standard errors,

d: L - Lower confidence interval 95%, U- upper confidence interval 95%

e: Significant indirect effects highlighted in bold

f: * *p* < .05, ** *p* < .001

## Discussion

This study asserts that some people engage with Facebook in problematic ways due to a reliance on social media in meeting their attachment needs, and that this engagement is accounted for in part by low self-esteem and high levels of psychological distress.

### Attachment anxiety and problematic Facebook use

Behaviourally, social comparison can involve compulsive scrolling through another person’s Facebook profile and timeline, whilst cognitively, it can include comparison of one’s abilities, and opinions to those of others [83]. While offline, the relationship between attachment anxiety and social comparison has been reported, it is argued that Facebook-specific social comparisons may be even more prevalent for individuals with high levels of attachment anxiety due to the increased availability of people with whom one can compare themselves with, in addition to the visibility of observable markers of popularity online, which can serve to heighten these tendencies. Previous research has identified links between attachment anxiety and Facebook surveillance [84], providing support for the preoccupation with others for those high in attachment anxiety within an online context.

Informed by their lived experiences, a desire for acceptance and a preoccupation with others is likely to be heightened during times of stress, as anxiously attached individuals strive to keep others close in order to restore a sense of security, thus offering clarification on the mediating role of psychological distress on this relationship. The mediating influence of low self-esteem may also be understood by considering the association between social comparison and self-enhancing motivations [85], in addition to a reduced certainty regarding self-concept in individuals with high levels of attachment insecurity [86].

Attachment anxiety also predicted engagement in impression management on Facebook. Given that users act as gatekeepers for information that is filtered to their online connections, impression management can be facilitated through the content made available in status updates, photo uploads, and personal biographies. This trend has been made evident in a range of studies, highlighting the frequency of impression management on SNSs [4, 87, 88]. When distressed, a desire for closeness and intimacy become heightened in those with high levels of attachment anxiety. However, their fear of rejection [40], results in conflicting drives, triggering sensitivity regarding how others will perceive them [89]. The creation of an online identity that is likely to be accepted and liked by others may be one strategy aimed at alleviating these concerns.

The current finding is partially consistent with the results of a previous study that reported links between attachment anxiety and sensitivity to social feedback on Facebook [54]. More direct support for this finding was recently provided by [66], who identified greater tendencies towards impression management via portrayal of a “false Facebook self”, in adults with high levels of attachment anxiety. In another study it was also suggested that this tendency is motivated by insecurity, when the authors reported that Facebook users with poor perceived relationship quality were more likely to make their relationship visible on their social networking profiles, by posting pictures of their partners or mentioning their partners in status updates [90].

In the context of the current study, self-disclosures referred to the over-sharing of personal information, both positive and negative, whilst on Facebook. A decision to disclose in this way might be considered to be evidence of proximity-seeking in individuals with high levels of attachment anxiety, the resultant desire of which is to receive attention and virtual contact from others, in the form of comments or ‘likes’. Empirical support for this assertion has been provided by [50] and [54] who reported links between attachment anxiety and attention seeking social media behaviour, and from [91], who reported that people disclose more on Facebook as a way of enhancing their popularity. With these findings in mind, the propensity for anxiously attached people to self-disclose can be better understood due to their need for acceptance and belonging. The decision to disclose on such a large network, instead of within a dyadic interplay, may also be accounted for by a lack of trust in others to meet ones emotional needs offline [92]. Perceived probability of responses within a large network may be a particular draw for such behaviour on Facebook, a finding supported by recent research which reported that the decision to disclose was related to the size and density of one’s social network [93].

Negative disclosures warrant additional mention, particularly considering previous findings that people use Facebook when in heightened emotional states [54, 94], and the current finding that psychological distress mediated the relationship between attachment anxiety and over-sharing on Facebook. Negative self-disclosures may therefore be partially explained by the difficulties in distress tolerance and inhibition of emotional spreading [95] in those high in attachment anxiety, and as a consequence, a greater tendency to display emotions and to seek support from others [96].

Intrusive use of Facebook in the current study focused on social and emotional consequences of use, and use of Facebook that impacted everyday functioning (e.g. sleep, work, study). Low self-esteem and high distress may trigger engagement in intrusive Facebook use for those high in attachment anxiety, arising from a perception that Facebook offers a greater sense of security that someone will be available to meet their needs online. This proclivity can be easily understood by considering the desire for high anxiety individuals, for human connection, and the capacity for Facebook to provide this, with few limits and restrictions. For example, there is ample opportunity to engage in digital connection with another person on Facebook, irrespective of time and location. A recent study by [97] indicated that Facebook users had an average of 150 online friends, despite reporting that only four of these were friends that they could rely on for support and comfort in offline contexts. The average number of Facebook friends held by the current sample was 370, therefore it is possible that access to a larger pool of people might further motivate people to engage intrusively with the site.

Previous research has identified a relationship between attachment anxiety and intrusive SNS use. A recent study for example found that adolescents with high levels of attachment anxiety were significantly more likely to engage in electronic intrusion, by using social media to monitor the activities and whereabouts of others, and pressure people for contact [98]. The researchers posited that the use of SNS’s may trigger a ‘cycle of anxiety’ for anxiously attached individuals, by simultaneously acting as a trigger for relationship anxiety and a tool for anxiety reduction.

### Attachment avoidance and problematic Facebook use

Contrasting with previous research reporting on a relationship between attachment avoidance and restrained use of Facebook [50], the current study found that attachment avoidance predicted intrusive Facebook use, resulting in social consequences for users of the site. This finding can be understood by considering the reluctance for intimacy and dependence noted in individuals with high levels of attachment avoidance [39, 99]. In this regard, Facebook may offer a suitable forum in which to have ones attachment needs met, since connections can be forged without the threat of closeness and intimacy. The finding that attachment avoidance predicted social consequences of intrusive Facebook use, but not risky/impulsive use, provides further support that engagement with Facebook may be a defensive strategy [49] aimed at creating emotional distance between these individuals and their offline connections, thus further maintaining their sense of behavioural independence [100]. The tendency for these individuals to suppress emotional distress due to a perceived sense that their vulnerable selves will not be acceptable to others, may explain why high levels of psychological distress and low self-esteem mediate the relationship between attachment avoidance and aspects of intrusive Facebook use. This assertion is consistent with research indicating that the Internet offers a virtual space where one can defensively retreat from painful emotions [101].

Though not originally hypothesized, the finding that attachment avoidance predicted engagement in impression management is consistent with recent research undertaken by [66], who found that individuals high in attachment avoidance were significantly more likely to engage in impression management on Facebook than those low in attachment avoidance. Additional support for these findings have been reported in offline contexts, where attachment security relative to insecurity has been associated with a reduced need to engage in defensive distortions regarding the self and less frequent appraisal regarding the similarity of the self in relation to others [102]. Low self-esteem and high distress may account for impression management, as a façade of social and emotional competence will serve to conceal vulnerabilities in those high in attachment avoidance. This assertion is consistent with previous research indicating that avoidantly attached individuals inflate their positive self-views when faced with threatening situations [102], and under-report feelings of intense emotion, despite the presence of physiological indicators of distress [103].

### Implications of the current findings

Hart and colleagues argued that for individuals with attachment insecurity, a reliance on Facebook may result in short lived feelings of well-being that reduce once people are offline [50]. The authors of this study question the ability of screen-based mediums such as Facebook to truly satisfy an individual’s fundamental attachment needs, particularly given the absence of touch, eye contact, voice prosody, and facial expression during online interactions. According to several researchers, it is these factors that are crucial in providing a sense of security, attunement, and safety to others during the development of attachment relationships [104–106]. For these reasons, the authors argue that Facebook cannot act as a suitable substitute for fundamental attachment needs, and thus reliance on these sites for these needs may lead to even greater interpersonal difficulties.

In considering the implications of the current findings, the authors suggest that they will be important for professionals involved in providing psychological and psychotherapeutic support to their clients. The authors recommend that information regarding social networking habits be gleaned as a matter of course during the assessment process, as this may help to unearth important precipitating and perpetuating factors when developing psychological formulations. For example, clients presenting with low self-esteem and low mood may be unaware that engagement with social comparison processes online might be maintaining their feelings of low self-worth, and as a consequence may fail to discuss this within the therapeutic context. Similarly, engagement in self-disclosure when in a heightened emotional state might further affect a person’s distress and self-esteem if they feel disappointed by the quantity and quality of the feedback that they receive from their online peers. Feedback regarding patterns of Facebook use may guide clinicians to discover more about the attachment orientation of their clients, thus providing them with additional information that can guide therapeutic intervention.

### Limitations and directions for future research

Though Bowlby [33, 35] has attested that attachment security remains relatively stable across the lifespan, it is not yet possible to link engagement in problematic Facebook use with early childhood experiences. According to [107] a range of factors can impact upon attachment patterns throughout the life span (cf. Life-Stress Model, Social-Cognitive Model, Individual Differences Model), and therefore future research interested in confirming the relationship between early childhood experiences and problematic Facebook use should endeavour to measure childhood attachment specifically. This may be achieved longitudinally or retrospectively via remembered parenting measures, which may provide some indication of childhood attachment via the quality of parent/child interactions.

As posited by [80], the rudimentary nature of simple mediation analyses can result in an oversimplification of the complexity of real-world relationships between variables. While psychological distress and self-esteem provide some explanation of the nature of the relationship between attachment and problematic Facebook use, there is huge scope to study this relationship further, focusing on a range of additional interpersonal factors relevant to attachment.

The cross-sectional nature of the current study limits the conclusions that can be drawn regarding the psychological outcomes associated with problematic Facebook use. However, due to the lack of authenticity associated with impression management [66], feelings of regret following disclosures [95], feelings of envy associated with social comparison [19], and the social and emotional impact of intrusive Facebook use, the authors consider it likely that the four patterns of Facebook use explored in this research may further impact the well-being of Facebook users. Utilisation of experience sampling methods in future research may serve to highlight potential causal relationships between variables, that may subsequently inform the completion of longitudinal research in this area.

Though the sampling method used in the current study was considered the most appropriate, given the exploratory nature of this research, use of non-probability sampling techniques does have the potential to introduce bias to study findings, which should be considered when interpreting the overall results of this research. That being said, the large sample size obtained may serve to enhance overall confidence with regards to the generalizability of the current findings.

The current study focused on four specific patterns of Facebook use that have been evidenced to be problematic insofar that their use has been linked to undesirable outcomes. There are a number of additional online ‘behaviours’ that can be examined within a similar theoretical framework. One example of this is Internet trolling – a recent phenomenon that refers to intentionally disruptive and harmful commentary carried out in a social setting on the Internet, that has no obvious purpose except to incite conflict in online environments. It is frequently encountered within SNSs and involves subjecting strangers to abuse and hateful messages. Given links with this behavior and attention-seeking [108], it may be worthwhile to consider within an attachment framework.

## Conclusions

The current study represents the first attempt, to the author’s knowledge, of applying attachment theory to understand adult engagement in problematic Facebook use. The findings suggest that Facebook may be used by some, in order to fulfill fundamental attachment needs, and that this use is accounted for, in part, by low self-esteem and difficulties in emotional regulation. While it is acknowledged that those high in attachment insecurity may derive some comfort and relief from using Facebook in these ways, the authors suggest that positive benefits may be short-lived, and that the nature of use could maintain distress and low self-esteem at their current levels, due to Facebook being a poor substitute for the gratification of highly significant human needs.

## Abbreviations

DASS: Depression, anxiety, and stress scale
ECR-R: Experiences in Close Relationships-Revised Questionnaire
INCOM: Iowa Netherlands Comparison Orientation Measure
IWM: Internal working model
NUIG: National University of Ireland, Galway
POFSS: Perception of false self-scale
PRIUSS: Problematic and risky internet use screening scale
RSES: Rosenberg self-esteem scale
SNS: Social networking site
SPSS: Statistical package for social sciences
